# Reclassifying Pseudopolyps in Inflammatory Bowel Disease: Histologic and Endoscopic Description in the New Era of Mucosal Healing

**DOI:** 10.1093/crocol/otz033

**Published:** 2019-10-15

**Authors:** Mona Rezapour, Maria Alejandra Quintero, Nidah S Khakoo, Daniel A Sussman, Jodie A Barkin, Jennifer Clarke, Tanya Varma, Amar R Deshpande, David H Kerman, Oriana Damas, Maria T Abreu

**Affiliations:** 1 Department of Gastroenterology and Hepatology, University of California Los Angeles, Los Angeles, California; 2 Department of Gastroenterology and Hepatology, University of Miami, Miami, Florida; 3 Department of Medicine, University of Miami, Miami, Florida; 4 Nebraska Food for Health Sciences, University of Nebraska-Lincoln, Lincoln, Nebraska; 5 Ameripath, Shreveport, Louisiana

**Keywords:** healed pseudopolyps, inflammatory bowel disease, inflammatory pseudopolyps

## Abstract

**Introduction:**

In this study, we identify the frequency of pseudopolyps (PPs) with normal histology and their association to surrounding tissue.

**Methods:**

Patients were enrolled in a study identifying endoscopic characteristics of PPs (n = 29) or were collected as part of our IBD biobank (n = 16). Statistical analysis included Stata v.15.0. chi-square and Student *t*-test.

**Results:**

A total of 45 patients with 117 PP biopsies were identified. More patients with healed PP were in endoscopic remission compared with those with inflammatory PP (82.6% vs 17.4%, respectively).

**Conclusion:**

This is the first study to find mucosal healing of PPs and its association with deep remission.

## Lay Summary

“Healed” pseudopolyps, which are projecting masses of scar tissue developed during the healing phase of IBD, were detected in patients with healed mucosa in the colon (endoscopic remission) more often than “inflammatory” pseudopolyps, which reflect active inflammation in the colon.

## INTRODUCTION

Inflammatory bowel disease (IBD), Crohn disease (CD), and ulcerative colitis (UC), are chronic, idiopathic inflammatory disorders affecting the gastrointestinal tract.^1^ Colorectal cancer risk is increased in individuals with long standing UC and CD colitis.^2^ Surveillance colonoscopy therefore becomes paramount for the detection of dysplasia.^2^ With the advent of newer endoscopic technologies including narrow band imaging (NBI) and chromoendoscopy (CE), detection and characterization of polypoid lesions has improved significantly.^3^

Pseudopolyps (PP) are characterized as islets of mucosa or mucosal tags that are presumed to form after severe ulceration and disrupted mucosal integrity.^4,5^ Granulation tissue can form in focal areas where there has been inflammation and inflammatory infiltration of the submucosa.^5,6^ Although PP can occur following many types of inflammatory insults, in IBD they represent polypoid protrusions of regenerating mucosa as compared with the surrounding mucosa.^6^ The surrounding mucosa could be inflamed or normal.^6^ Endoscopically, PP can be sessile, pedunculated, or filiform.^7^ They can be single or multiple and range in size including giant PP so called because of their large size of greater than 15 mm mimicking villous adenomas or carcinoma.^7,8^ Although most PP are not dysplastic, historically, patients with PP were thought to be at an increased risk for colitis-associated cancer (CAC).^9,10^ However, recently a multicenter retrospective cohort found no association between PPs and CAC.^11^ The reported prevalence of PP is about 10%–20% in patients with UC and CD^12^ and is similar in both sexes.^5^ The incidence of PP peaks in IBD patients in their second to fourth decade of life.^5^ Furthermore, the prevalence rises with extensive colitis and longer duration of active disease.^13^

We previously published a study that examined endoscopic characteristics of inflammatory PPs. We used a Delphi method to identify endoscopic features suggestive of inflammatory PP histology to build a consensus prediction system.^14^ All the PPs that were evaluated endoscopically were also sampled for histology. In our initial publication, we characterized only the endoscopic features of inflammatory PP as defined by pathology. In the current study, we describe the frequency of PP with normal colonic histology, termed healed PP, compared with inflammatory PP, the relationship between PP histology and surrounding inflammation, and the relationship of PP histology and concurrent medications. We hypothesized that patients in deep remission were more likely to have PPs that had normal colonic histology compared with patients with active inflammation.

## METHODS

### Study Design and Population

We included patients who underwent colonoscopy at our center and had the presence of PPs on examination. We obtained data of patients from our prior study that described the endoscopic characteristics of inflammatory PPs^14^ and also from patients with PPs on colonoscopy that were captured in our UM IBD Biorepository. The patients included from the biorepository were those with PPs that were biopsied and that also had biopsies of the surrounding tissue. In addition, we only included patients whose biopsy reports were available on our electronical medical records (EMR) for evaluation. All patients had a standard bowel preparation for colonoscopy. For procedures completed between June 2010 and February 2013, the Olympus adult CF-180 and pediatric PCF-180 colonoscopes (Olympus Corporation, Shinjuku, Japan) were used. For procedures between June 2013 and April 2017, the Olympus adult CF-190 and pediatric PCF-190 colonoscopes (Olympus Corporation) were used. All colonoscopies were performed for clinical purposes at the treating physicians' discretion, including evaluation of response to medical therapy, routine surveillance for dysplasia, and evaluation of symptoms such as diarrhea or rectal bleeding.

Endoscopic inflammation was graded using standard endoscopic scoring systems, including Mayo score for UC^15^ and the Simple Endoscopic Score for CD.^16^ These scores were entered at the time of colonoscopy in our IBD database. In addition, the endoscopist assigned a rating of never inflamed, uninflamed, mild, moderate, or severely inflamed for each segment of bowel that was also entered into the IBD database.

Directed biopsies were performed using a multibite forceps (Boston Scientific, Natick, MA). Biopsies of colonic tissue adjacent to PP were taken. All histology slides were reviewed by dedicated gastroenterology pathologists. The pathologists were not blinded to the endoscopic findings.

### Data Collection

Clinical and demographic data were captured from the IBD intake research questionnaire. The following baseline and clinical data were collected from the IBD intake questionnaire and confirmed with the electronic health records: birthdate, sex, age at the time of study, type of IBD [UC, CD, or indeterminate colitis (IC)], year of diagnosis, and duration of disease. In addition, the maximum extent of luminal disease at diagnosis and at the time of colonoscopy was obtained from the electronic medical record and from the colonoscopy report, respectively. Furthermore, we collected exposure to any previous medications including 5-aminosalicylates, immunomodulators, methotrexate, steroids, and biologics (including infliximab, adalimumab, certolizumab, golimumab, ustekinumab, natalizumab, vedolizumab, and experimental biologics). We also documented therapy at the time of colonoscopy.

### Endoscopic Assessment of PP Features

For the 29 patients (59 PPs) that were part of the PP endoscopic study, each polyp was examined endoscopically in a protocolized way with white light (WLE), NBI, and CE. Features consistent with PPs and not adenomas included fibrinous cap, surface friability and ulceration, an appendage-like appearance, central dark area surrounded by a clear lighter area (halo sign) on CE, white exudate on a smooth surface with sharp borders and a clustering of polyps.^14^ In addition, the endoscopist described the Kudo pit pattern classification of the PP.^17^ Under WLE, PPs were graded based on certain suggestive features that included elongated shape, fibrin cap, and other.^14^ “Other” features include smooth, flat, and normal appearing mucosal surface.^14^

### Histology of PP

A “healed PP” was defined by the absence of acute or chronic inflammation in the lamina propria and without the presence of granulation tissue or ulceration. An “inflammatory” PP was defined as one with either active (neutrophil predominant) or chronic (lymphocyte predominant) inflammation in the lamina propria and/or the presence of granulation tissue or ulceration. The location of the PP was also documented as rectosigmoid, descending colon, splenic flexure, transverse colon, hepatic flexure, ascending colon, or cecum.

### Outcomes and Analyses

Our objectives were to determine the clinical implications of endoscopically appearing PP with normal histology (“healed PPs”). More specifically, we examined the association between healed PPs and adjacent luminal inflammation, overall colon inflammation, type of IBD (UC or CD) and medications. We then examined whether there are endoscopic features that would allow endoscopists to distinguish between a healed PP and an inflammatory PP, and whether the presence of one healed PP is associated with the presence of other healed PPs.

We used chi-square tests to examine the relationship between PP histology (healed or inflammatory) and categorical values, including IBD type, presence of adjacent colonic inflammation, presence of endoscopic inflammation in the entire colon, location of PP in the colon, endoscopic description of PP, and type of medication. We used binomial logistic regression to examine the influence of colonic inflammation and biologic medication use on PP histology (healed vs not healed). Pearson correlation was used to examine the association between a healed PP and concomitant presence of other healed PPs. Statistical analysis was done using Stata v.15.0.

### Study Oversight

The “real-time diagnosis of PPs during colonoscopy using noninvasive advanced endoscopic techniques—a prospective study” and the “Inflammatory bowel disease (IBD) center clinical phenotype database and specimen collection” were approved by the University of Miami Institutional Review Board (IRB). Patients in the prospective study consented to the IRB-approved protocol. And all patients in the University of Miami biorepository are consented as well.

## RESULTS

### Patient Characteristics and Relationship to PP Histology

A total of 45 patients with IBD and PPs were identified between 2010 and 2017. Of these, 29 were prospectively identified and the other 16 retrospectively. There was a total of 117 PP biopsy samples examined in this study. Of the 117 samples, 36 (30.77%) were inflammatory PPs and 81 (69.23%) were healed PPs. Of the 45 patients, 18 (40.0%) patients had CD, 26 (57.77%) patients had UC, and 1 (2.22%) patient had IC (Table 1). There were 28 (62%) females and 17 (38%) males. Healed PPs were more often found in patients with UC (74.1%) versus CD (23.5%), *P* = 0.006. In patients with CD, 50% had ileocolonic disease followed by isolated colonic disease (44.4%) and ileal disease (5.6%). UC patients were more likely to have pancolonic disease (88.5%) followed by left-sided disease (11.5%) (Table 1).

**TABLE 1.** Demographic Data

Demographics	N (%)
Gender	
1. F	28 (62.2%)
2. M	17 (37.8%)
Age	44.18 ± 14.7
Disease phenotype	
1. CD	18 (40.0%)
2. IC	1 (2.2%)
3. UC	26 (57.8%)
Location of disease—CD	
1. Colonic	8 (44.4%)
2. Ileal	1 (5.6%)
3. Ileocolonic	9 (50.0%)
Location of disease—IC	
1. Colonic	1 (100%)
Location of disease—UC	
1. Left-sided	3 (11.5%)
2. Pancolonic	23 (88.5%)

### PP Histology Tracks With Surrounding Histologic Inflammation

We asked whether the histology of the PP related to the histologic activity of disease in the surrounding mucosa. For all patients included in this analysis, biopsies were taken in the same segment of colon as the PP. We found that in patients with healed PPs, 64 (79.0%) had inactive colitis in the area adjacent to the PP and 17 (21.0%) had active inflammation in the area adjacent to the PP (*P* = 7.72 × 10^−9^, Table 2). These data suggest that PP histology represents part of the generalized inflammatory process.

**TABLE 2.** PP Histology in Association With Disease Activity

Pseudopolyp Histology	Inflammatory PPs, n (%)	Healed PPs, n (%)	*P*
*Total number*			
Endoscopic activity	36 (30.8%)	81 (69.2%)	0.010
1. Inactive colitis (remission)	8 (17.4%)	38 (82.6%)	
2. Active colitis	27 (39.1%)	42 (60.9%)	
Area adjacent to PP			7.72 × 10^−9^
1. Inactive colitis	8 (11.1%)	64 (88.9%)	
2. Active colitis	28 (62.2%)	17 (37.8%)	
Location of PP			0.203
1. Rectum	4 (40%)	6 (60%)	
2. Rectosigmoid	3 (60%)	2 (40%)	
3. Sigmoid	15 (39.5%)	23 (60.5%)	
4. Descending colon	6 (31.6%)	13 (68.4%)	
5. Splenic flexure	1 (50%)	1 (50%)	
6. Transverse colon	4 (22.2%)	14 (77.8%)	
7. Hepatic flexure	0 (0.0%)	3 (100%)	
8. Ascending colon	2 (13.3%)	13 (86.7%)	
9. Cecum	0 (0.0%)	6 (100%)	
Presence of dysplasia at time of study			0.441
1. Dysplasia present	5 (35.7%)	9 (64.3%)	
2. Dysplasia absent	27 (30%)	63 (70%)	
Presence of dysplasia on follow-up colonoscopy			0.676
1. Dysplasia present	2 (33.3%)	4 (66.7%)	
2. Dysplasia absent	25 (33.8%)	49 (66.2%)	

In terms of location of PP, there was no statistically significant difference between the locations of healed versus inflammatory PPs (*P* = 0.122). This may be due to the fact that both inflammatory and healed PPs were mainly located in the rectosigmoid regardless of histology (45.7%) (Table 2).

Patients with one healed PP on colonoscopy were more likely to have other healed PPs identified during the same colonoscopy than inflammatory PPs (*r* = 0.48, *P* = 0.012). Further, 60% of patients with a healed PP had all other PPs that were healed (60% all healed PP vs 40% mixed inflammatory and healed, *P* = 0.023).

We also wanted to evaluate whether presence of dysplasia at the time of study colonoscopy was an important consideration when evaluating inflammatory versus healed PPs. We did not find a statistically significant difference between healed versus inflammatory PPs in terms of whether dysplasia was present on the study colonoscopy (*P* = 0.44) as few patients had dysplasia (13.5%) at the time of study colonoscopy (Table 2). There was no dysplasia present in any of the PPs themselves.

Lastly, we looked at the presence of dysplasia on any follow-up colonoscopies. Again, we did not find a statistically significant difference between healed versus inflammatory PPs in terms of presence of dysplasia on follow-up colonoscopy (*P* = 1.0). However, the small number of patients included in the analysis prevents statistical evaluation. Few patients with either healed or inflammatory PPs had dysplasia on follow-up colonoscopy (7.5%) (Table 2).

### The Endoscopic Appearance of the PP Relates to Histology

**FIGURE 1.**
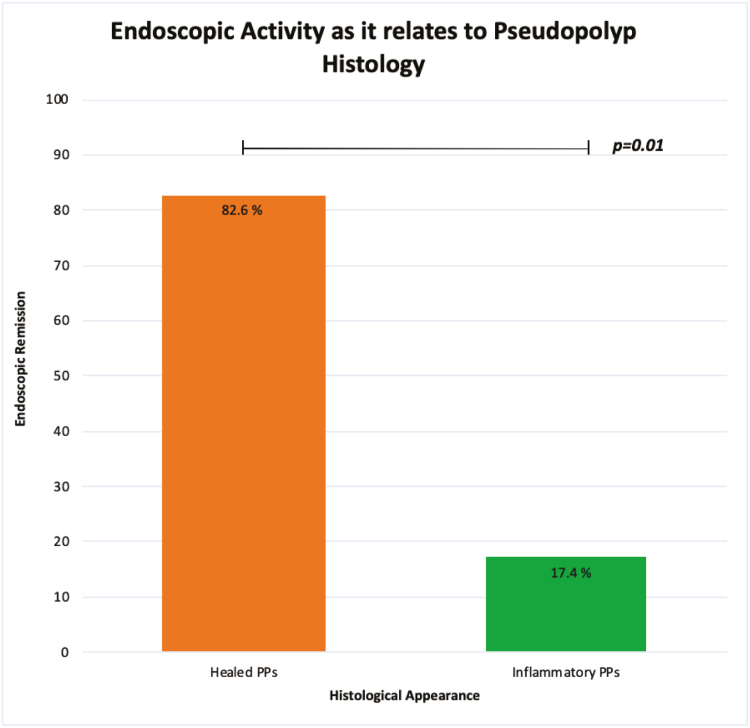
Global endoscopic activity as it relates to PP histology.

We next compared the histology of the PP as it related to the global endoscopic disease activity. We found that patients with healed PPs were in endoscopic remission 82.6% of the time as compared with 17.4% of patients with inflammatory PPs (*P* = 0.01) (Fig. 1). Thus, PP histology tracks with endoscopic activity.

**FIGURE 2.**
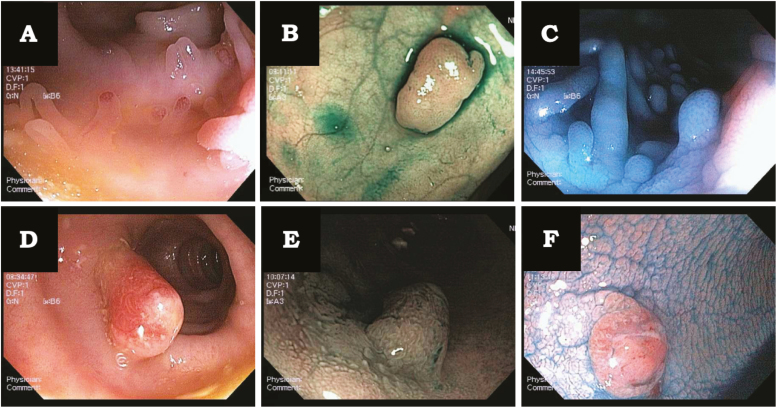
Endoscopic appearance of healed PPs. A, Healed PP with smooth surface and elongated shape on white light endoscopy (WLE). B, Healed PP with dot vascular pattern on narrow band imaging (NBI). C, Healed PP with Kudo pit pattern I on chromoendoscopy (CE). D, Inflammatory PP with fibrin cap on WLE. E, Inflammatory PP with gyrus pattern on NBI. F, Inflammatory PP with Kudo pit pattern type IV on CE.

In a prospective study of 29 patients with PP, we performed a protocolized endoscopy and examined PPs (n = 59) in high definition WLE, NBI, and dye spray with indigo carmine (CE) (Fig. 2). In an earlier published study, we used a Delphi method to describe endoscopic features of inflammatory polyps but did not describe healed PPs.^14^ The feature noted in WLE suggestive of healed PPs were elongated shape and “other” as compared with suggestive feature of inflammatory PPs which was fibrin cap (*P* = 0.028) (Table 3). Under NBI, we examined vascular patterns of PPs endoscopically. We found that a vascular pattern of dots was associated with healed PPs (*P* = 0.012). About 87.9% of healed PPs had a dot vascular pattern. We next evaluated the PPs by CE based on the Kudo's pit pattern classification as types I, II, IIIs, IIIL, IV, Va, and Vn. We found that 81.1% of healed PPs had Kudo pit pattern I (*P* = 0.042) (Table 3).

**TABLE 3.** Endoscopic Features of PP in Relation to Histology

Endoscopic Features	Inflammatory PPs, n (%)	Healed PPs, n (%)	*P*
Suggestive features on WLE			0.028
1. Elongated shape	7 (21.9%)	25 (78.1%)	
2. Fibrin cap	10 (58.8%)	7 (41.2%)	
3. Other*	1 (11.1%)	8 (88.9%)	
Vascular pattern on NBI			0.012
1. Dots	4 (12.1%)	29 (87.9%)	
2. Fibrin cap	5 (50%)	5 (50%)	
3. Gyrus	4 (66.7%)	2 (33.3%)	
4. Halo	2 (40%)	3 (60%)	
Kudo pit pattern on CE			0.042
1. I	7 (18.9%)	30 (81.1%)	
2. II	0 (0.0%)	3 (100%)	
3. IIIL	6 (54.5%)	5 (45.5%)	
4. IIIs	0 (0.0%)	1 (100%)	
5. IV	3 (75%)	1 (25%)	
6. V	1 (100%)	0 (0.0%)	

*“Other” features include smooth, flat, and normal appearing mucosal surface.

### More Patients on Biologics and Combination Therapy Had Healed PP

We evaluated the relationship of PP histology to current medication therapy, duration on biologic therapy, and duration on immunomodulators. Looking at the medications that patients were taking at the time of study, there was a statistical significance (*P* = 0.005) between the medication and the histology of PPs. In patients with healed PPs, 55.3% were treated with combination therapy or biologics as compared with 44.7% of patients with inflammatory PPs. However, we performed a multi regression analysis to see if the significance seen was due to medication effect or rather the amount of inflammation in the adjacent area. On multiregression analysis, we noted that the effect of medication therapy was no longer statistically significant (*P* = 0.128) but inflammation in the adjacent area was noted to be significant (*P* = 5.6 × 10^−9^). Therefore, when taking into account presence of colonic inflammation as a covariable, biologic use was no longer predictive of PP histology. Thus, anything that results in mucosal healing influences PP histology rather than the type of medication.

Prior exposure to immunomodulators or biologics was numerically more frequent in patients with healed versus inflammatory PPs. About 67.7% of patients with healed PPs had prior exposure to immunomodulators as compared with 32.3% of patients with inflammatory PPs (*P* = 0.433). About 61.4% of patients with healed PPs had exposure to biologic therapy as compared with 38.6% of patients with inflammatory PPs (*P* = 0.056) (Table 4).

**TABLE 4.** PP Histology and Medication Use

Medical Therapy	Inflammatory PPs, n (%)	Healed PPs, n (%)	*P*
Medication therapy			0.005
1. None	2 (66.7%)	1 (33.3%)	
2. Steroids	2 (100%)	0 (0.0%)	
3. Mesalamines	6 (15%)	34 (85%)	
4. Immunomodulators	5 (20%)	20 (80%)	
5. Biologics	12 (46.2%)	14 (53.8%)	
6. Combination	9 (42.9%)	12 (57.1%)	
Any prior exposure to immunomodulators	20 (32.3%)	42 (67.7%)	0.433
Any prior exposure to biologics	22 (38.6%)	35 (61.4%)	0.056

## DISCUSSION

The current study was undertaken to highlight the differences in PP histology and the implications for surveillance colonoscopies in IBD. The majority of patients with PPs had UC and healed PP architecture. In patients with healed PP, the mucosa surrounding the PP was also not inflamed. Furthermore, patients with healed PP were more likely to be in endoscopic remission globally and patients with one healed PP on colonoscopy were more likely to have all the other sampled PPs also exhibit healed histology. This is the first study to examine mucosal healing in PPs and to find an association between deep remission and healed PPs. Although this study does not have long-term follow-up for dysplasia, we may extrapolate the results of Christensen et al^18^ that healed colitic mucosa is much less likely to be associated with dysplasia long term.

In our study, we found that most patients with healed PPs were in endoscopic remission. This finding is notable as, historically, PPs were found to be independent predictors of colorectal carcinoma in IBD patients (CAC).^19–21^ A more recent multicenter retrospective study by Mahmoud et al^11^ did not, however, find an association between development of CRC and PPs; PPs were thought to be a surrogate for the inflammatory burden rather than a risk factor for CRC. The results from our study suggest that most modern day PPs have a healed phenotype and may be the explanation why PPs are no longer harbingers of increased CAC risk. Perhaps in part due to a small sample size, we found very few patients with dysplasia regardless of PP histology. Mahmoud et al^11^ specifically controlled for predictors of CAC development including histologic inflammation which could account for the lack of association between development of CAC and PPs. Furthermore, PPs were not stratified into healed versus inflammatory and therefore it remains possible that patients with healed PP have a lower risk of CRC development as compared with patients with inflammatory PPs.

A foundational study by De Dombal et al^22^ determined that patients with PP are more likely to have had a previous severe flare. Over half of the patients in this cohort in 1956 who underwent colectomy had PPs.^22^ A meta-analysis by Maggs et al^6^ showed that about 70% of patients with PPs have extensive colitis and none had isolated proctitis. Furthermore, PPs can be complicated by obstruction, bleeding, iron-deficiency anemia and protein-losing enteropathy and therefore, along with concerns for development of dysplasia, surgical resection used to be a common modality for the treatment of PPs.^6,23^ Mahmoud et al^11^ most recently corroborated this notion that IBD patients with PP are more likely to undergo colectomy and had a significantly shorter time to colectomy. However, the findings from our study that endoscopic remission is associated with healed PPs and that the majority of patients with one healed PP are more likely to have other healed PPs in the same colonoscopy may dissuade gastroenterologist from recommending colectomy as a management strategy for PPs.

The literature is lacking in terms of endoscopic characterization of PP mucosa. When we evaluated the endoscopic characteristics of PPs and compared them to the histologic findings, we found that healed PPs were more likely (87.9% of the time) to demonstrate a “dot-like” vascular pattern as compared to inflammatory PPs. Rastogi et al^24^ described the vascular pattern of tubular adenomas and hyperplastic polyps on NBI and found that hyperplastic polyps displayed a circular pattern with dots with a sensitivity of 86% and specificity of 97%. We also found that healed PPs are more likely to display a Kudo pit pattern type I, which according to Kudo et al^17^ is a nonneoplastic pit pattern. These findings can help the endoscopist in identifying healed PPs during colonoscopy as the identification can help avoid multiple biopsies and polypectomies of PPs during colonoscopy.

There are limitations to our study. Twenty-nine of the patients were part of a protocolized PP endoscopic study with the rest of the patients from our IBD biorepository. For the patients in the biorepository, the reporting of PPs was part of the standard template of endoscopy reporting in our endoscopy reporting software but not protocolized. For this reason, the endoscopic characteristics are only included for the 29 patients in the initial PP study. We only included patients that had PP biopsies and biopsies of flat mucosa from the same segment of colon. All of the colonoscopies included in this study were performed by dedicated IBD physicians at our institution. These clinicians are experienced in the use of CE for dysplasia surveillance as well. Nevertheless, there could be a lack of uniformity between physicians. Although all histology slides were reviewed by dedicated gastroenterology pathologists, they were not blinded to the endoscopic findings. This has the potential for bias even though we attempted to mitigate this bias by having more than one dedicated pathologist review the slides. Our sample size was small because we only included patients with matched biopsies. Many more patients had PPs described that were not biopsied and thus not included in the current analysis. Several patients initiated medical therapy before our electronic medical records and the exact time on medications was not available. In the future, larger studies are needed to validate the findings in our study. Long-term follow-up is also needed to determine if healed PPs have a more favorable course than inflammatory PPs.

In conclusion, we were able to confirm mucosal healing of PPs especially in patients who were in deep remission. Given that PPs may have a risk of dysplasia, these data add to the principle that histologic remission is important to minimize progression to dysplasia. In addition, based on the high degree of concordance between healed PP and certain endoscopic features, endoscopists may not need to biopsy healed PPs.

## Notes

Supported by: Research funding from Takeda (A.R.D), grant K23KD117054 (O.D.) funded by National Institute of Health (NIH), and grant/research from Prometheus, Takeda, Eli Lilly Pharmaceuticals, Pfizer (M.T.A.).

Conflict of interest: D.A.S.: Olympus Corporation, Grant identifier: Industry, Clinicaltrials.gov identifier: NCT01557387. Principal investigator: D.A.S. Date of award: August 18, 2011–August 17, 2015. Advisory board for Exactsciences, Inc. A.R.D.: consulting, GI Health; board member, American Board of Internal Medicine. D.H.K.: Advisory Board/Consulting—Abbvie, Cleveland Clinic, PRIME CME. M.T.A.: Consultant—Abbvie, Prometheus, Takeda, Janssen, Focus Medical Communications, Celgene Corporation, SERES, Eli Lilly Pharmaceuticals, Pfizer, Shire, Roche, Boehringer Ingelheim, Amgen, Gilead, Allergan, Landos Biopharma, Nestec Ltd, Switzerland. Speaker's Bureau—Takeda, Focus Medical Communicaions, Cornerstones Health, Imedex, Vindico Medical Education. The other authors declared that they do not have any conflicts of interest.

## References

[1] Baumgart DC, Sandborn WJ Inflammatory bowel disease: clinical aspects and established and evolving therapies. Lancet. 2007;369:1641–1657. 17499606 10.1016/S0140-6736(07)60751-X

[2] Laine L, Kaltenbach T, Barkun A, et al; SCENIC Guideline Development Panel. SCENIC international consensus statement on surveillance and management of dysplasia in inflammatory bowel disease. Gastroenterology. 2015;148:639.e28–651.e28. 25702852 10.1053/j.gastro.2015.01.031

[3] Rutter MD, Saunders BP, Wilkinson KH, et al Most dysplasia in ulcerative colitis is visible at colonoscopy. Gastrointest Endosc. 2004;60:334–339. 15332019 10.1016/s0016-5107(04)01710-9

[4] Goldgraber MB Pseudopolyps in ulcerative colitis. Dis Colon Rectum. 1965;8:355–363. 5830658 10.1007/BF02627260

[5] Jalan KN, Walker RJ, Sircus W, et al Pseudopolyposis in ulcerative colitis. Lancet. 1969; 2:555–559. 4185531 10.1016/s0140-6736(69)90260-8

[6] Maggs JR, Browning LC, Warren BF, Travis SP Obstructing giant post-inflammatory polyposis in ulcerative colitis: case report and review of the literature. J Crohns Colitis. 2008;2:170–180. 21172208 10.1016/j.crohns.2007.10.007

[7] Orlawska J, Jarosz D, Bielecki K, Wejman J Diffuse filiform polyposis of the colon in Crohn's disease. Acta Pathol Microbiol Et Scan. 1996;101:323–325. 10.1111/j.1699-0463.1996.tb00692.x 8619920

[8] Kelly JK, Langevin JM, Price LM, et al Giant and symptomatic inflammatory polyps of the colon in idiopathic inflammatory bowel disease. Am J Surg Pathol. 1986;10:420–428. 3717497 10.1097/00000478-198606000-00007

[9] Magro F, Gionchetti P, Eliakim R, et al; European Crohn's and Colitis Organisation [ECCO]. Third European evidence-based consensus on diagnosis and management of ulcerative colitis. Part 1: definitions, diagnosis, extra-intestinal manifestations, pregnancy, cancer surveillance, surgery, and ileo-anal pouch disorders. J Crohns Colitis. 2017;11:649–670. 28158501 10.1093/ecco-jcc/jjx008

[10] Annese V, Daperno M, Rutter MD, et al; European Crohn's and Colitis Organisation. European evidence based consensus for endoscopy in inflammatory bowel disease. J Crohns Colitis. 2013;7:982–1018. 24184171 10.1016/j.crohns.2013.09.016

[11] Mahmoud R, Shah SC, Ten Hove JR, et al; Dutch Initiative on Crohn and Colitis. No association between pseudopolyps and colorectal neoplasia in patients with inflammatory bowel diseases. Gastroenterology. 2019;156:1333–1344.e3. 30529584 10.1053/j.gastro.2018.11.067 PMC7354096

[12] Ooi BS, Tjandra JJ, Pedersen JS, Bhathal PS Giant pseudopolyposis in inflammatory bowel disease. Aust N Z J Surg. 2000;70:389–393. 10830609 10.1046/j.1440-1622.2000.01826.x

[13] Rutter M, Saunders B, Wilkinson K, et al Severity of inflammation is a risk factor for colorectal neoplasia in ulcerative colitis. Gastroenterology. 2004;126:451–459. 14762782 10.1053/j.gastro.2003.11.010

[14] Sussman DA, Barkin JA, Martin AM, et al Development of advanced imaging criteria for the endoscopic identification of inflammatory polyps. Clin Transl Gastroenterol. 2015;6:e128. 26583503 10.1038/ctg.2015.51 PMC4816089

[15] Naganuma M, Ichikawa H, Inoue N, et al Novel endoscopic activity index is useful for choosing treatment in severe active ulcerative colitis patients. J Gastroenterol. 2010;45:936–943. 20401498 10.1007/s00535-010-0244-2

[16] Daperno M, D'Haens G, Van Assche G, et al Development and validation of a new, simplified endoscopic activity score for Crohn's disease: the SES-CD. Gastrointest Endosc. 2004;60:505–512. 15472670 10.1016/s0016-5107(04)01878-4

[17] Kudo S, Tamura S, Nakajima T, et al Diagnosis of colorectal tumorous lesions by magnifying endoscopy. Gastrointest Endosc. 1996;44:8–14. 8836710 10.1016/s0016-5107(96)70222-5

[18] Christensen B, Hanauer SB, Erlich J, et al. Histologic normalization occurs in ulcerative colitis and is associated with improved clinical outcomes. Clin Gastroenterol Hepatol. 2017;15:1557.e1–1564.e1. 28238954 10.1016/j.cgh.2017.02.016 PMC5618439

[19] Velayos FS, Loftus EV Jr, Jess T, et al Predictive and protective factors associated with colorectal cancer in ulcerative colitis: a case–control study. Gastroenterology. 2006;130:1941–1949. 16762617 10.1053/j.gastro.2006.03.028

[20] Baars JE, Looman CW, Steyerberg EW, et al The risk of inflammatory bowel disease-related colorectal carcinoma is limited: results from a nationwide nested case–control study. Am J Gastroenterol. 2011;106:319–328. 21045815 10.1038/ajg.2010.428

[21] Rutter MD, Saunders BP, Wilkinson KH, et al Cancer surveillance in longstanding ulcerative colitis: endoscopic appearances help predict cancer risk. Gut. 2004; 53:1813–1816. 15542520 10.1136/gut.2003.038505 PMC1774334

[22] De Dombal FT, Watts JM, Watkinson G, Goligher JC Local complications of ulcerative colitis: stricture, pseudopolyposis, and carcinoma of colon and rectum. Br Med J. 1966;1:1442–1447. 5933046 10.1136/bmj.1.5501.1442 PMC1844640

[23] Anderson R, Kaariainen IT, Hanauer SB Protein-losing enteropathy and massive pulmonary embolism in a patient with giant inflammatory polyposis and quiescent ulcerative colitis. Am J Med. 1996;101:323–325. 8873495 10.1016/S0002-9343(96)00201-X

[24] Rastogi A, Bansal A, Wani S, et al Narrow-band imaging colonoscopy—a pilot feasibility study for the detection of polyps and correlation of surface patterns with polyp histologic diagnosis. Gastrointest Endosc. 2008;67:280–286. 18155210 10.1016/j.gie.2007.07.036

